# A Modern Approach to the Treatment of Traumatic Brain Injury

**DOI:** 10.3390/medicines11050010

**Published:** 2024-04-30

**Authors:** Marat Syzdykbayev, Maksut Kazymov, Marat Aubakirov, Aigul Kurmangazina, Ernar Kairkhanov, Rustem Kazangapov, Zhanna Bryzhakhina, Saule Imangazinova, Anton Sheinin

**Affiliations:** 1 Department of Hospital Surgery, Anesthesiology and Reanimatology, Semey Medical University, Semey 071400, Kazakhstan; marat.syzdykbayev@smu.edu.kz; 2 Department of General Practitioners, Semey Medical University, Semey 071400, Kazakhstan; maxut.kazymov@smu.edu.kz; 3 Department of Pediatric Surgery, Semey Medical University, Semey 071400, Kazakhstan; aubakirov54@mail.ru; 4 Committee for Medical and Pharmaceutical Control of the Ministry of Health of the Republic of Kazakhstan for East Kazakhstan Region, Ust-Kamenogorsk 070004, Kazakhstan; 5 Pavlodar Branch of Semey Medical University, Pavlodar S03Y3M1, Kazakhstan; kairkhanov67@mail.ru (E.K.); rustem.kazangapov@bk.ru (R.K.); 6 Department Psychiatry and Narcology, Semey Medical University, Semey 071400, Kazakhstan; bryzhahina.janna@yandex.kz; 7 Department of Therapy, Astana Medical University, Astana 010000, Kazakhstan; dr_iss@mail.ru; 8 Sagol School of Neuroscience, Tel-Aviv University, Tel-Aviv 69978, Israel

**Keywords:** traumatic brain injury, brain, trauma, therapy

## Abstract

**Background:** Traumatic brain injury manifests itself in various forms, ranging from mild impairment of consciousness to severe coma and death. Traumatic brain injury remains one of the leading causes of morbidity and mortality. Currently, there is no therapy to reverse the effects associated with traumatic brain injury. New neuroprotective treatments for severe traumatic brain injury have not achieved significant clinical success. **Methods:** A literature review was performed to summarize the recent interdisciplinary findings on management of traumatic brain injury from both clinical and experimental perspective. **Results:** In the present review, we discuss the concepts of traditional and new approaches to treatment of traumatic brain injury. The recent development of different drug delivery approaches to the central nervous system is also discussed. **Conclusions:** The management of traumatic brain injury could be aimed either at the pathological mechanisms initiating the secondary brain injury or alleviating the symptoms accompanying the injury. In many cases, however, the treatment should be complex and include a variety of medical interventions and combination therapy.

## 1. Introduction

The approach to brain injury has evolved with advances in medical science and the application of evidence-based medicine. Over the past 20 years, more than 30 traumatic brain injury (TBI) guidelines have been developed and updated by various organizations [1,2]. However, there is currently no ultimate and effective guideline or treatment protocol for TBI. Thus, the positive effects observed in randomized clinical trials (RCTs) of various pharmacological treatments of severe TBI were small, typically with <10% difference between groups [3,4].

Effective treatment requires the identification of key therapeutic targets. There are two general approaches to the development of TBI management today. The first, the “traditional” approach is mainly based on neuroprotection and utilizes an identification and targeting of the key mechanisms involved in the development of secondary injury, whether it be severe or mild TBI. In this traditional approach, treatment is usually initiated as soon as possible after the injury since delaying neuroprotective therapy is thought to reduce its effectiveness. Traditional therapy often targets key initiators of the secondary injury cascade. The second, an “alternative” approach being explored in clinical trials for patients with mild TBI is symptomatic rather than mechanistic treatment aimed at symptoms such as headache, sleep disturbances, vestibular/oculomotor disturbances, post-traumatic stress disorder, cognitive dysfunction, or other secondary consequences [5].

This review discusses several current research concepts and strategies for TBI management, both “traditional” and “alternative”. The general overview of the discussed strategies and methods of TBI treatment is outlined in Table 1 below.

## 2. Concepts and Strategies for TBI Therapy

Symptomatic treatment is often used in some emergency cases of severe TBI. Based on clinical or physiological manifestations, particular forms of therapy (such as hyperosmolar agents, barbiturates, hypothermia, and craniectomy) are used to alleviate intracranial hypertension [6,7,8]. However, in finding an effective treatment for severe TBI the main research attention has always been focused on novel neuroprotective agents [9,10]. Thus, using a traditional, “mechanistic” approach, Kochanek et al. [9], Mondello et al. [11], and Yang et al. [12] have attempted to develop new neuroprotective therapies for TBI treatment. Operation brain trauma therapy (OBTT) is just one approach to establishing a TBI screening consortium. The OBTT uses a scoring matrix that includes 22 points per animal model assessed on several parameters including motor, cognitive, histological, and serum biomarkers [11]. Using the OBTT, a total of 12 drugs have been tested, namely nicotinamide, erythropoietin, cyclosporin A, simvastatin, levetiracetam, glibenclamide, Kollidon@VA-64, amantadine, minocycline, axiostatin (E64d), and P7C3-A20. Two drugs, levetiracetam and glibenclamide, appeared to be the most promising. Thus, levetiracetam improved multiple outcomes in two experimental models of TBI, namely fluid percussion brain injury (FPI) and controlled cortical impact (CCI) [13], while glibenclamide displayed relatively stable benefit in CCI [14,15]; however, the above benefits were largely limited to the particular experimental model.

The goal of novel approaches to TBI management is to identify and develop robust therapy in a pre-clinical study, determine the optimal dosage and treatment regimens, and implement the most effective methods in medical practice. Therefore, optimal communication between pre-clinical and clinical studies appears to be essential for the successful development and implementation of new methods of TBI management and improved patient outcomes.

## 3. Secondary Brain Injury in TBI

Despite significant improvements in resuscitation after TBI, one of the inevitable consequences of neurotrauma is secondary brain injury. Direct mechanical impact can cause force dynamics to be transmitted through the dura mater, cerebrospinal fluid, and brain parenchyma. This can cause axons to become dislocated, cell membranes to break down, and fluid to shift, leading to swelling of the brain tissue. This primary trauma eventually develops into a secondary trauma response [16]. Although currently there is no FDA-approved drug to prevent or treat damage secondary to neurotrauma [17], understanding the mechanisms of pathophysiology of secondary brain injury is important for the successful treatment of traumatic brain injury.

Biochemical, cellular, and physiological events occurring during primary injury often develop into delayed and prolonged secondary injuries that can last from hours to years. Secondary brain injury can cause many harmful effects such as cerebral edema, blood–brain barrier (BBB) disruption, increased intracranial pressure, metabolic dysfunctions, excitotoxicity, oxidative and cellular apoptosis, or necrosis, ultimately leading to neurological dysfunctions [18].

A cascade of secondary damage typically unfolds over time, ranging from minutes to days after the initial damage. It may be influenced by many factors such as systemic hypotension, hypoxemia, increased intracranial pressure, and metabolic imbalance [10,19]. In addition, the severity of the secondary injury and its impact on disease outcome depends on various factors, including the nature and extent of the primary injury, as well as the effectiveness of medical interventions aimed at mitigating the progression of the damage [18,20,21,22,23,24].

Secondary damage from neurotrauma is associated with endoplasmic reticulum (ER) and mitochondrial dysfunction, as well as oxidative stress [10,25,26,27,28]. Stress on these organelles triggers a cascade of events that ultimately activate neuroinflammatory pathways [28,29]. Signs of neuroinflammation or CNS immune response begin to develop soon after the initial injury. Some obvious changes include ventricular enlargement, edema, and white and/or gray matter atrophy, while microscopic apoptosis, autophagy, axonal damage, and necrosis are visualized [30,31].

## 4. Emergency Care for TBI

### 4.1. Head Position

Lifting the head of a patient with a traumatic brain injury usually has quick consequences. The intracranial pressure (ICP) is reduced due to the displacement of cerebrospinal fluid (CSF) from the intracranial space and increasing of venous outflow [32]. Although mean carotid pressure decreases with head elevation, ICP decreases while cerebral blood flow (CBF) does not change [33].

### 4.2. Airway Management

#### 4.2.1. Tracheostomy

Tracheostomy is often performed on TBI patients, mainly because of many associated beneficial effects, such as protection of airways from aspiration, enhanced comfort of the patient, decreased requirement for sedation, improved pulmonary hygiene, and more effective secretion suction [34]. Tracheostomy decreases the duration of stay in the intensive care unit, as well as the number of days on an artificial ventilation machine [34].

There is ongoing debate about the benefits of early tracheostomy over late tracheostomy [35,36]. Early tracheostomy could be associated with more ventilator-free days [36,37]. Thus, several recent studies demonstrated that early tracheostomy significantly decreases both pulmonary morbidity and critical care resource utilization [37,38,39,40,41,42].

#### 4.2.2. Hyperventilation

Hyperventilation reduces ICP by lowering the arterial partial pressure of carbon dioxide (PaCO_2_), which subsequently leads to vasoconstriction. This sequence of events ultimately leads to a decrease in brain blood volume [43]. Prophylactic hyperventilation is generally not recommended since vasoconstriction reduces the CBF. As a result of vasoconstriction, focal foci of ischemia may occur, especially in areas of preserved autoregulation [44].

#### 4.2.3. Ventilator-Associated Pneumonia

Early onset ventilator-associated pneumonia (EOVAP) is considered a common complication in TBI patients, with a frequency of occurrence of 24–60% [45,46]. EOVAP could impair cerebral oxygenation and its occurrence is a factor in worsening neurological outcomes [46]. The risk factors of EOVAP in TBI patients are not fully determined, and the effects of various pre-hospital methods of airway management techniques on EOVAP occurrence in this population have not yet been investigated. However, there is some evidence that pre-hospital airway management does not significantly affect the occurrence of EOVAP in patients with severe TBI [47]. Similarly, despite the increased duration of mechanical ventilation and prolonged stay in the intensive care unit, pre-hospital airway management appears to have no significant effect on mortality or long-term neurological outcomes [47].

### 4.3. Prevention of Seizures

Current recommendations for TBI treatment state that prophylactic administration of antiepileptic drugs for one week could be used to prevent early seizures. However, to date, there has been no proven benefit in preventing late seizures after TBI, and therefore antiepileptic therapy is usually discontinued after 7 days [48].

A large prospective randomized controlled study found no difference in the incidence of early post-traumatic seizures between a group of patients treated with phenytoin and another group treated with levetiracetam [49].

### 4.4. Sedation and Induced Coma

One of the last steps of maximal treatment is to put a patient into a drug-induced coma, usually by infusion of benzodiazepines such as midazolam or infusion of barbiturates such as pentobarbital. Benzodiazepines and barbiturates exert their action by a significant reduction of the metabolic demands in the brain. Prophylactic use of barbiturates for seizure suppression is usually not recommended. However, the administration of barbiturates could be used in severe refractory intracranial hypertension after exhaustion of maximum medical and surgical therapy targeting reduction of intracranial pressure [43].

Reducing stress and adrenocortical response is also considered an important component of TBI treatment [50]. By reducing cerebral metabolism and oxygen consumption in a dose-dependent manner [51], sedatives can reduce metabolic stress in acutely injured brain tissue and lead to a decrease in the ICP. Achieving an adequate level of sedation is of paramount importance, since it minimizes the length of hospital stay, the number of days on a ventilator, and the frequency of delirium and promotes early mobilization [52]. The Brain Trauma Foundation (BTF) recommendations [6] regarding the use of sedatives and analgesics are as follows:

1. Administration of barbiturates to suppress seizures and as a prophylaxis for intracranial hypertension is not recommended (grade IIB).
2. High doses of barbiturates are recommended to control intracranial pressure refractory to maximum standard surgical and medical treatments while maintaining hemodynamic stability.
3. Although propofol can be used to control intracranial pressure, it is not recommended for reducing mortality or six-month outcomes.

### 4.5. Hypothermia

Oxidative stress is believed to be a secondary effect of TBI. Therapeutic hypothermia in infants and children has been shown to reduce oxidative damage [53].

There are several plausible mechanisms by which hypothermia may mitigate the effects of TBI, including a decrease in intracranial pressure, a decrease in the innate inflammatory response, and a decrease in the cerebral metabolic rate [49,54].

The multicenter, non-blinded RCT Eurotherm3235 (2015) is the largest study of hypothermia in patients with intracranial hypertension (>20 mmHg) after TBI. This study has demonstrated that therapeutic mild hypothermia [48,49,50,51] (32–35 °C) combined with standard therapy to reduce ICP resulted in a slightly increased mortality rate and poor functional outcomes compared with patients receiving standard therapy alone. In addition, an unfavorable association between hypothermia and the progression of multiple organ failure has been observed [55,56].

It should be stressed that deep hypothermia (below 30 °C) does not appear to have a beneficial effect on TBI, while mild to moderate hypothermia (32–35 °C) appears to be neuroprotective [57,58].

Hypothermia (32–34 °C) reduces cerebral metabolic rate, preserves tissues in the face of metabolic problems, and reduces intracranial pressure. There is insufficient evidence to support prophylactic hypothermia; although not currently a standard therapy, it may be an option for a multimodal approach, as supported by TBI guidelines [59]. The use of hypothermia is not a standard recommendation in the management of patients with TBI [6]. Patients with TBI treated with hypothermia show little improvement in outcomes. However, in patients with severe TBI, fever should be actively controlled [60].

### 4.6. Blood Pressure and Cerebral Perfusion Pressure (CPP)

For optimal perfusion of organs and tissues, the systolic blood pressure should be maintained at a range of 110–130 mm Hg while diastolic blood pressure should not be allowed to decrease below 60 mm Hg. Increased mortality (about 35%) of TBI patients with systolic blood pressure (SBP) ≤ 85 mm Hg has been reported, compared to only 6% mortality in patients with higher SBP [61].

For resuscitation, intravenous administration of fluids and vasopressors should be used [62]. The ideal intravenous fluid for TBI patients remains a matter of debate (please see “Fluid management” below). However, hypotonic fluids should be avoided to prevent volume overload and cerebral edema [63,64].

The 4th edition of the BTF manual recommends [4]:

1. To reduce mortality and improve the outcomes (level III), maintain SBP at ≥100 mm Hg for patients aged 50 to 69 years or at a level ≥110 mm Hg or higher for patients aged 15 to 49 or older than 70.
2. The recommended CPP target for survival and good outcomes is 60 to 70 mm Hg. The optimal CPP may depend on the patient’s autoregulatory status (level IIB).
3. Aggressive attempts to maintain CPP above 70 mm Hg with fluids and vasopressors should be avoided.

### 4.7. Fluid Management

Available evidence suggests that the volume of fluid administered, rather than the choice of the fluid itself, plays an important role in outcomes after TBI [54,63]. Saline is the most common crystalloid solution used in TBI patients, but Ringer’s lactate could be used as an alternative. Crystalloid solutions generally do not expand well, and about 70–80% of their infused volume usually reaches the interstitial space within 20 min of infusion, contributing to general systemic tissue edema. During the breaching of the blood–brain barrier (BBB), the significant passive spread of the crystalloid solution to the interstitium of the brain can occur and this, in turn, can exacerbate the cerebral edema and increase the intracranial pressure, especially when using hypotonic solutions. In addition, the infusion of large volumes of saline can lead to unfavorable hyperchloremic metabolic acidosis, which is dangerous in TBI. Balanced crystalloid solutions can be a better alternative to saline [65].

Hypertonic crystalloid solutions, such as hypertonic saline, glycerol, urea, sorbitol, and mannitol are often used to reduce brain swelling in TBI. The hypertonic solutions decrease intracranial pressure and promote the shrinkage of the brain tissue by establishing a strong trans-epithelial osmotic gradient and directing the water flow from the brain tissue to the intravascular compartment. However, some of the above solutions have significant side effects. For example, the use of urea could be associated with nausea, vomiting, diarrhea, hemoglobinuria, and rebound intracranial hypertension [66]. Sorbitol and glycerol are often associated with a significant increase in blood glucose levels which could be additionally harmful to TBI patients [66]. Hypertonic saline has been widely used for the treatment of TBI since 1919 [67]. The solution is available in different concentrations (3.0–23.4%); however, there is no conclusion on the effectiveness and safety of particular concentrations in TBI treatment. Hypertonic saline in the 3.0–7.5% range could be infused without dilution or in combination with isotonic crystalloids or colloids for hypovolemic shock resuscitation [68]. Mannitol appears to be effective in reversing acute brain swelling; however, its effectiveness in the ongoing management of TBI remains unclear [69]. The use of mannitol has been associated with hypotension [66,70,71], rebound increase in intracranial pressure [72], and renal toxicity [73]. Overall, mannitol treatment was reported to be associated with a detrimental effect on mortality when compared to hypertonic saline [69].

Colloid solutions do not appear to provide additional benefits, and the Saline versus Albumin Fluid Evaluation SAFE) study found increased mortality in patients treated with albumin compared with saline [74]. In many patients (9–23%), TBI is linked to acute renal injury and frequently results in increased mortality [75]. Additionally, it has been discovered that administration of colloids raises the risk of acute kidney injury and increases the need for renal replacement treatment in severe TBI. According to a recent assessment, colloids are not more effective than crystalloids in terms of overall mortality, particularly when it comes to patients who have had surgery, trauma, or burns [76]. However, colloid administration in patients with preserved renal function may be considered [76].

### 4.8. Tranexamic Acid

Administration of a hemostatic agent, tranexamic acid, is necessary for the prevention of hemorrhages in TBI. Currently, available data from the CRASH-3 study suggest that the administration of tranexamic acid to patients with mild or moderate TBI resulted in a reduction of mortality by 20% [77]. Also, the meta-analysis of Yokobori et al. [78] suggested that treatment with tranexamic acid displayed a trend toward reduced mortality from head injuries in patients with severe TBI without a significant incidence of thromboembolic complications. However, Bossers et al. [79] demonstrated that the pre-hospital administration of tranexamic acid was associated with a higher mortality rate in patients with severe TBI, especially isolated TBI.

The meta-analysis of Huang et al. [80] demonstrated that administration of tranexamic acid during the initial 3 h after the trauma may reduce mean hemorrhage volume. However, the reduction of mean hemorrhage volume was not observed when the drug was administered more than 3 h after trauma [80]. Therefore, the timing of tranexamic acid administration appears to be one of the crucial factors affecting its hemostatic effect. 

Thus, the results of current studies do not provide a clear answer to the question of whether the use of tranexamic acid reduces mortality and morbidity rates, especially in patients with severe TBI. However, treatment with tranexamic acid could be effective in patients with mild or moderate TBI. 

## 5. Surgical Interventions for TBI

Decompressive craniectomy (DC) is an effective, lifesaving option for lowering intracranial pressure in TBI patients [81]. Most DCs are performed via a standard trauma flap shaped like a reverse question mark (RQM) [81]. As an alternative to an RQM, the safer and more effective Ludwig Kempe hemispherectomy incision (Kempe) could be used [82]. It has been shown that DC can be lifesaving for patients with TBI with a decrease in mortality [82]. The BTF guidelines recommend a larger frontotemporoparietal decompressive craniectomy, rather than a smaller one, to reduce mortality and improve neurological outcomes [6].

One of the consequences of DC is the need for cranioplasty (replacement of a bone defect with an artificial plate). Currently, there is no consensus on the timing of cranioplasty, cranioplasty material (e.g., titanium, polyethylene, methyl methacrylate, and hydroxyapatite), or the use of autologous bone [83].

Cisternostomy is a novel surgical strategy for severe TBI management. Several reports demonstrated promising results with the use of cisternostomy compared to the traditional methods of TBI treatment [84,85,86].

## 6. Pharmacological Therapy of TBI

### 6.1. Corticosteroids

Corticosteroids reduce the production of CSF and exert antiedematous and anti-inflammatory effects in TBI. The first large-scale pharmacological trial of TBI was the randomization trial of corticosteroids after a major head injury (MRC corticosteroid randomization after significant head injury (CRASH), 2004). The patients with moderate to severe TBI were given a 48-h infusion with high doses of corticosteroids (methylprednisolone) or a placebo. In this multicenter study, 10,008 patients from 49 countries participated. The primary outcome of the study was 2-week mortality, which was higher in the treatment group (21.1%) than in the placebo group (17.9%). After 6 months of follow-up, there were 173 additional deaths in the treatment group (1248 vs. 1075) [87]. These results show there is no reduction in mortality with corticosteroid administration in the 2 weeks after head injury. 

### 6.2. Progesterone

Following the failure of corticosteroids in TBI, pre-clinical animal studies have focused on the early administration of progesterone, a potent neurosteroid synthesized in the central nervous system. It has been demonstrated that the administration of progesterone reduces neuronal loss, cerebral edema, and behavioral disturbances after experimental TBI. However, large double-blind, placebo-controlled, multicenter phase III RCTs (SYNAPSE and PROTECT III) failed to demonstrate a significant positive effect of progesterone on patient mortality and functional outcomes, which led to the end of the enthusiasm generated by two previous single-center clinical trials [4,88,89].

### 6.3. Erythropoietin

Erythropoietin (EPO) is a glycoprotein that regulates hematopoiesis in the bone marrow, which is naturally produced in the kidneys after hypoxic stimulation. Animal studies have demonstrated that EPO can neutralize neuronal apoptosis, reduce the inflammatory response, and act as a neurotrophic factor, thereby alleviating the consequences of secondary brain damage in TBI [90].

A meta-analysis of six RCTs involving 1041 patients which examined the results of EPO treatment after acute (moderate or severe) TBI demonstrated that EPO administration significantly reduced mortality but failed to reduce the incidence of adverse outcomes and did not improve the functional outcome [90]. There were no significant differences in complication rates, including deep vein thrombosis, between the EPO treatment and the control groups [90]. In general, the use of EPO in severe TBI appears to be controversial since some individual studies demonstrated benefits on functional outcomes while other studies failed to find any significant difference in outcomes [3,88,89,91].

### 6.4. Amantadine

Amantadine is a dopamine agonist used in Parkinson’s disease. Amantadine can be distributed to the frontal lobes and acts as an N-methyl-D-aspartate (NMDA) receptor antagonist. It has been suggested that the administration of amantadine increases the external dopamine levels in the striatum and results in antagonism of NMDA receptor function [92], thereby protecting neurons from glutamate excitotoxicity in the acute phase of TBI. It has been shown that at doses of 200–400 mg/day, amantadine can improve arousal and cognition in TBI patients [93]. Other studies compared the effect of amantadine versus placebo; the results suggest that amantadine accelerated the pace of functional recovery in patients with severe TBI [94,95].

### 6.5. N-Acetylcysteine

It has been shown that in various animal models, N-acetylcysteine (NAC) has a significant neuroprotective effect by reducing the consequences of secondary neuronal damage. Rat models have confirmed the beneficial antioxidant properties of NAC in the treatment of brain injury [96]. It has been proposed that NAC exerts its neuroprotective effect through increasing levels of GSH, a combination of L-glutamic acid, L-cysteine, and glycine in the brain [97]. NAC administration maintains high levels of GSH in the brain, which acts as a free radical scavenger and as an antioxidant [96,97,98].

The effectiveness of NAC in treating patients with moderate blast-induced TBI was assessed in a double-blind, placebo-controlled human trial [99]. After receiving 2 g NAC twice a day for the first 4 days, the treatment group was given 1.5 g NAC twice a day for the next 3 days. After seven days of treatment, all patients were evaluated for headache, dizziness, memory loss, hearing loss, sleep difficulties, and neurocognitive impairment. Seven days after treatment, patients who received NAC within 24 h of the injury reported a significant improvement in these symptoms. In addition, in the treatment group, the chances for recovery were 86% [99].

### 6.6. Minocycline

It has been demonstrated that the tetracycline antibiotic minocycline (MINO), both alone and in combination with NAC, has neuroprotective qualities. The effectiveness of MINO in treating neurological problems resulting from traumatic brain injury has been demonstrated in rat models [100,101]. The memory and cognition of animals were enhanced in a mildly controlled cortical model by combined treatment with MINO + NAC, which also restored white matter by shielding oligodendrocytes [100].

### 6.7. Phenserine

In the treatment of TBI, the activity of phenserine reduces neuroinflammation, facilitates amyloid deposition, prevents apoptosis, and mitigates many different secondary injury mechanisms [102,103,104,105].

### 6.8. Calcium Channel Blockers

It has been shown that nimodipine, an L-type calcium channel blocker, improves outcomes for patients who have spontaneous subarachnoid hemorrhage [106]. A thorough study, however, found no statistically significant difference in death and morbidity rates between TBI patients treated with nimodipine and placebo [107].

Administration of an N-type calcium channel blocker ziconotide (SNX-111) between 15 min and 10 h after TBI has been shown to improve mitochondrial function in animals [108,109]. Another N-type calcium channel blocker SNX-185 has been reported to be neuroprotective when administered directly to hippocampal CA2 and CA3 24 h after TBI in rats [110]. More clinical research is required to determine the effectiveness and safety of calcium channel blockers in TBI treatment.

### 6.9. Antioxidants

The immunosuppressant cyclosporin A, a potent regulator of mitochondrial permeability transition pores (mPTPs), has been shown to have a neuroprotective effect in experimental TBI models [111]. However, a small randomized clinical trial of cyclosporin A in TBI unexpectedly demonstrated no improvement in neurological outcomes and biochemical parameters in patients with severe TBI [112].

### 6.10. Beta-Blockers

The benefits of early beta-blocker therapy in TBI have been demonstrated in several retrospective and prospective observational studies demonstrating a positive impact of beta-blockers on clinical outcomes and survival [113,114,115,116,117]. It has been hypothesized that the adrenergic storm caused by the primary stroke may exacerbate secondary brain injury through the mechanisms of cerebral vasoconstriction and subsequent ischemia [118,119]. Thus, hyperadrenergic activity is associated with an increased risk of death due to the exacerbation of secondary brain injury and induction of extracranial multiorgan dysfunction, especially cardiovascular, pulmonary, and inflammatory consequences [120,121].

Since the cerebral perfusion and subsequent oxygen delivery to the cerebral tissue are impaired due to the catecholamine-induced cerebral vasoconstriction [118,119], the use of beta-blockers to improve the brain environment in this context is based on physiological principles.

The early oral administration of beta-blocker propranolol in patients with isolated severe TBI results in improved survival and functional outcomes up to 6 months post-injury [122]. The above results provide support for the routine administration of beta-blockers as a part of standardized neuroresuscitation protocol. A meta-analysis by Ding et al. [123] demonstrated that the administration of beta-blockers after the TBI was safe and effective. Nevertheless, more studies are needed to investigate the effectiveness and safety of beta-blockers in TBI treatment.

### 6.11. Metformin

An antihyperglycemic agent metformin has been used clinically for decades as a treatment for diabetes. However, in addition to glucose regulation, metformin also stimulates neurogenesis [124] and has potent anti-inflammatory properties [125,126,127]. The above properties make metformin an interesting candidate for the treatment of CNS injuries. Indeed, several recent studies demonstrate the neuroprotective effects of metformin in various models of CNS injury [128,129,130].

Metformin significantly improves cognition after controlled cortical injury in mice, showing improved spatial learning and nest-building [131]. In addition, injured animals treated with metformin demonstrated increased ramification of microglial processes, indicating a reduction in neuroinflammation. Finally, in vitro, metformin treatment increased the activation of partitioning defective (Par1), a family of Ser/Thr kinases playing a key role in synaptic plasticity and neuroinflammation [131]. However, the improvement in behavioral outcomes of TBI by metformin treatment remains unclear, and the molecular mechanisms of metformin action are not well understood.

### 6.12. Cerebrolysin

The use of multimodal neuropeptide cerebrolysin in TBI treatment has been proposed by several studies [132,133,134,135,136]. It has been shown that cerebrolysin helps prevent secondary injury cascade by controlling oxidative stress, microglial activation, inflammation, and blood–brain barrier dysfunction [137]. However, the research on the effects of cerebrolysin in inoperable patients with severe TBI remains limited.

The recent meta-analysis demonstrated that intravenous administration of cerebrolysin significantly improved functional outcomes in patients with TBI measured by the Glasgow Outcome Scale and the modified Rankin Scale scores [138]. The cerebrolysin-treated group of inoperable patients with severe TBI demonstrated higher rates of recovery, as evidenced by an improvement in Glasgow Outcome Scale score and a shorter duration of stay in the hospital than the patients of the control group [139].

### 6.13. Vitamin D

Infectious complications, such as pneumonia and sepsis, are common in brain trauma patients and result in significant mortality in these patients. In addition, these patients have a high prevalence of vitamin D deficiency [140]. Since vitamin D deficiency is also associated with serious complications such as coma, slow neurological recovery, and persistent critical illness polyneuropathy in TBI patients [141,142,143], treatment with vitamin D could be of great importance in patients with TBI. Thus, Arabi et al. [144] suggest that vitamin D administration significantly reduces the mortality rate and inflammation in TBI patients.

## 7. Regenerative Treatment

### 7.1. Neurotrophic Factors

The post-traumatic fate of neurons and glial cells can be influenced by neurotrophic factors, such as vascular endothelial growth factor (VEGF), brain-derived neurotrophic factor (BDNF), nerve growth factor (NGF), basic fibroblast growth factor (bFGF), and epidermal growth factor (EGF). Administering of these growth factors following traumatic brain injury may enhance the recovery and neurological outcome [145,146].

### 7.2. Suppression of RhoA GTPase

Recent studies demonstrated that the small GTPase RhoA is essential for mediating the effects of molecules acting against axonal regeneration on damaged myelin and glial scar. The ADP-ribosylation of Rho proteins by the exoenzyme C3-transferrase from *Clostridium botulinum* may block the downstream signaling of this cascade. As a result of the ADP-ribosylation, the axon regeneration is facilitated [147,148].

Due to the wide variety of cellular functions that C3-transferase has in promoting CNS regeneration, combined therapy using this enzyme and other neuroprotective medications may have an additive impact in the treatment of TBI [149]. Given the parallels between TBI and spinal injuries, it can be presumed that the benefits of C3-transferase treatment seen in spinal injuries could also apply to TBI, although the importance of C3-transferase in experimental models of TBI has not yet been established. 

### 7.3. DNA Vaccine

DNA vaccination is a novel and relatively simple method of inducing an immunological response. Recent studies have suggested that a DNA vaccine encoding the inhibitory epitopes of myelin-associated proteins may be a promising approach to repairing damaged CNS [150,151].

### 7.4. Protein S100B

The S100B protein is a calcium-binding protein produced by glial cells. The S100B protein has been detected in serum after breaching the BBB following brain injury [152]. It has been shown that the administration of S100B exhibits a dose-dependent dual effect on neurons. In small doses, S100B acts as a neurotrophic neuroprotective factor. However, at high doses, S100B increases neuroinflammation and impairs neuronal survival [153,154].

### 7.5. Overcoming the Glial Scar

Significant activation of chondroitin sulfate proteoglycans (CSPGs) such as neurocan, phosphacan, versican, and NG2 in the glial scar contributes to impaired axonal regeneration after CNS injury. Administration of CSPG-cleaving enzyme chondroitinase ABC reduces CSPG levels and cavitation at the site of injury within 24 h [155]. Overexpression of chondroitinase ABC in transgenic mice also improved axon regeneration in astrocytic scars [156]. Thus, the administration of inhibitory molecules to the glial scar may represent promising targets for accelerating regeneration in TBI.

### 7.6. Stem Cell Therapy

Losses of neurons and glia are the main signs of CNS damage. Therefore, the replacement of these cells represents a viable approach to therapy. Injection of mesenchymal stem cells in an acute TBI model reduced the expression of various proinflammatory cytokines and chemokines, such as IL-1β, IL-6, TNF-α, CCL2, CCL11, and CXCL [157]. In addition, recent studies have shown that stem cells improve functional recovery after TBI due to regenerative and neuromodulatory properties [158].

Among various types of stem cells, numerous studies suggested the excellent neuroprotective properties of neural stem cell (NSC)transplantation. By mitigating neuroinflammation and stimulating regenerative processes (i.e., enhancing neurogenesis, angiogenesis, and plasticity), NSCs may support functional recovery after acute TBI [159,160,161].

Guo S. et al. (2019) demonstrated that when administered intranasally, the exosomes derived from mesenchymal stem cells (MSC-Exo) can cross the blood–brain barrier and migrate to the injured area of the spinal cord [162]. MSC-Exo loaded with phosphatase and tensin homolog small interfering RNA (ExoPTEN) significantly enhanced axon growth and neovascularization while reducing microgliosis and astrogliosis. Intranasal therapy with ExoPTEN may also partially improve structural and electrophysiological function and, most importantly, significantly induce functional recovery in rats with complete spinal cord interruption. The results show that intranasal ExoPTEN can be used in a clinical setting to speed up the recovery of patients with spinal cord injuries [162].

Overall, stem cell therapy has been demonstrated as a useful tool that can reduce the effects of TBI. These treatments appear to be safe and have been shown to improve neurological and motor function in TBI patients.

### 7.7. Nanoparticles

The clinical use of pharmacological treatment in TBI has been hampered by limited possibilities for drug delivery to the brain. In addition, the retention of pharmacological substances in the brain is also limited. Therefore, the use of nanoparticles as drug carriers has received increasing attention in the last few years. Encapsulation of drugs in micro- or nanoparticles appears to be a promising way to ensure sustained and controlled drug delivery in TBI [163]. Both natural and synthetic polymers have been successfully used as drug carriers and depots. The polymers used as carriers share the following common characteristics: biocompatible, biodegradable, generally inert, and capable of attaching to or encapsulating small molecules and proteins [164].

Recently, it has been shown that treatment of a TBI rat model with cerium oxide (CeO_2_) nanoparticles can significantly reduce brain damage by restoring cognitive abilities and enhancing antioxidant properties [165]. Another study reported that the administration of immunomodulatory nanoparticles markedly improves motor impairment and reduces inflammation and edema in a mouse model of TBI [166].

Recently, the combination of stem cell therapy with nanoparticle delivery gained increasing research attention. Thus, Narouiepour [167] reported that combining human fetal brain-derived stem cells with niosomal nanoparticles can improve functional recovery and reduce neuroinflammation in a TBI model [167]. In addition, Zhu et al. demonstrated the possibility of labeling human stem cells with superparamagnetic iron oxide nanoparticles and tracking them using magnetic resonance imaging (MRI) [168]. A 34-year-old patient in this study had left temporal lobe craniocerebral damage. The exposed neural tissue was removed during emergency surgery, and the cells were cultured in order to select stem cells. The cultured stem cells were then treated by a contrast agent containing superparamagnetic iron oxide nanoparticles. Next, the autologous cultured stem cells were stereotactically injected into the damaged brain area. The MRI signal change one-week post-implantation was correlated with the growth and accumulation of cells surrounding the lesion. During the second and third weeks, the signal around the lesion grew, indicating that the stem cells moved from the main injection sites to the edge of the injured tissue [168].

Currently, due to relatively low reproducibility, poor scalability, and high cost, the use of nanoparticles in TBI treatment is somewhat limited. Therefore, to increase the clinical application of the nanoparticles, the development of scalable, low-cost synthesis is required.

## 8. Treatment Based on Physical Principles

### 8.1. Hyperbaric Oxygen Therapy (HBOT)

Currently, hyperbaric oxygen therapy (HBOT) is considered one of the most important clinical treatments for TBI. Harch et al. [169] treated 16 patients with TBI, post-TBI syndrome, and post-traumatic stress disorder (PTSD) utilizing 40 sessions of HBO at 1.5 ATA of oxygen/60 min for 30 days, which significantly improved symptoms, neurological examination results, comprehensive IQ test results, and cognitive function. In addition, Geng et al. [170] demonstrated that HBO can suppress the activation of inflammatory signals, thereby alleviating TBI. In severe TBI, HBOT reduces mortality and improves functional outcomes [171,172].

In several randomized and randomized controlled trials, HBO at 1.5 ATA of oxygen displayed statistically significant symptomatic and cognitive improvements in patients with mild traumatic brain injury and persistent post-concussion syndrome. Positive and negative results were observed at lower and higher doses of oxygen and pressure [173]. Overall, these studies indicate the successful use of HBOT in intensive care for the treatment of patients with TBI.

### 8.2. Non-Invasive Brain Stimulation

To date, several technologies of non-invasive brain stimulation have been developed. The most common technologies are transcranial magnetic stimulation (TMS) and transcranial direct current stimulation (tDCS). Repetitive transcranial magnetic stimulation (rTMS) is a painless, non-invasive, easy-to-administer treatment with few adverse reactions. It has been shown that rTMS has a specific effect on the rehabilitation of patients with TBI [174,175].

Neville et al. [176] conducted a double-blind randomized controlled trial in 36 patients with TBI who were randomly and equally divided into two groups. Ten sessions of high frequency rTMS (10 Hz) over the left dorsolateral pre-frontal cortex (DLPFC) were administered to TBI patients in the treatment group, while pseudostimulation was administered to TBI patients in the sham group. To evaluate immediate and delayed effects, patients completed cognitive evaluations one week prior to, one week following, and three months following rTMS. The findings suggest that rTMS can enhance post-TBI depression and cognitive function [176].

Recently, Dhaliwal etc. [177] has reported that brain stimulation may have a potential impact on the treatment of TBI and that rTMS can be used as an effective treatment for some post-TBI symptoms, such as depression, tinnitus, and neglect.

## 9. Complementary Medicine

Complementary and integrative medicine approaches, including acupuncture and herbal medicine, are often used to complement the limitations of conventional medicine [178,179], improving efficacy and sometimes reducing side effects even in the treatment of traumatic brain injury [180,181].However, currently available data suggest insufficient evidence to recommend these complementary methods of TBI treatment in clinical practice.

## 10. Mild TBI Management

The majority of TBIs (up to 90%) are mild [182]. Although some studies reported a relatively low mortality rate of patients with mild TBI [183], these patients remain at increased risk of mortality in the 5 years post-injury [184]. In addition, many survivors of mild TBI experience symptoms for years. For mild TBI, the World Health Organization (WHO) lists the following diagnostic criteria: “(i) one or more of the following: confusion of disorientation, loss of consciousness for ≤30 min, post-traumatic amnesia (PTA) for <24 h and/or other transient neurological abnormalities, such as focal signs, seizure and intracranial lesion not requiring surgery; AND (ii) a Glasgow Coma Scale (GCS) score of 13–15 after 30 min post head injury or later upon presentation for health care. These manifestations of mild TBI must not be due to drugs, alcohol, medications, caused by other injuries or treatment for other injuries (e.g., psychological trauma, language barrier or coexisting medical conditions) or caused by penetrating craniocerebral injury” [185,186].

Many patients with mild TBI are often discharged from the hospital during the first 24 h post-injury provided there are no serious complications or worsening of the status of the patient. Some mild TBI symptoms may appear immediately after the trauma, while others may not appear for hours or days after the injury. Moreover, some patients may remain generally asymptomatic for 1–4 months. Symptoms generally improve over time and many patients feel better within a couple of weeks.

Symptoms of mild TBI are different from person to person. The most common include headache, dizziness, fatigue, sleep disturbances (insomnia, hypersomnia, nightmares), attentional difficulties, memory problems, depression, anxiety, photo- and phono-sensitivity, irritability, and depersonalization [186,187]. The long-term consequences of mild TBI may include cognitive deficit [188], dementia [189], Alzheimer’s disease [190,191], and a high risk of developing neurological disorders, including epilepsy [192] and stroke [193].

The management of mild TBI in many cases could be symptomatic. However, careful consideration of the severity of the initial injury was recommended [194]. While the WHO definition of mild TBI stated above applies to mild TBI, the possibility for a more severe TBI should be assessed and excluded by Mayo criteria [194]. Imaging techniques (MRI, CT scan) should be implemented, especially when suspecting post-traumatic amnesia (PTA), bleeding, or expanding lesions [186]. The proper assessment of individual neurological and neuropsychiatric symptoms should determine individualized treatment. At early stages and in patients with somatic complaints, the intervention should address the treatment of the somatic symptoms. In patients with cognitive or emotional symptoms, neuropsychological help is advised [186,187]. Thus, for example, antidepressants as well as psychotherapy could be administered to patients with depression and anxiety; cognitive behavioral therapy could be effective in reducing concentration and memory difficulties [186]. The overall approach to the treatment of mild TBI should be, therefore, individualized and highly dependent on the severity and character of the injury.

## 11. Conclusions

TBI is a major global health problem and one of the main priorities in modern critical care. Despite the lack of an effective treatment for TBI recovery, efforts to develop therapeutic strategies for TBI recovery have been continuously undertaken over the past few decades. Today, standard medical and surgical interventions always play a significant role in the emergency care of patients with TBI.

There are several promising areas of research aimed at optimizing neuroresuscitation protocols, developing an evidence base for surgical intervention, and promising neuroprotective pharmacotherapy. Given the heterogeneity of the pathology and the fact that one intervention method cannot affect all involved pathological mechanisms, the importance of personalized medicine and combination therapy in the field of TBI cannot be overestimated.

## Figures and Tables

**Table 1:** Overview of the modern approach to the treatment of traumatic brain injury.

Strategies of TBI Management	Specific Methods of TBI Treatment
Emergency care of TBI	Head position Airway management Tracheostomy Hyperventilation Ventilator-associated pneumonia Prevention of seizures Sedation and induced coma Hypothermia Blood pressure and cerebral perfusion pressure Fluid management Tranexamic acid
Surgical interventions for TBI	Decompressive craniectomy Kempe hemispherectomy incision Cisternostomy
Pharmacological therapy of TBI	Corticosteroids Progesterone Erythropoietin Amantadine N-acetylcysteine Minocycline Phenserine Calcium channel blockers Antioxidants Beta-blockers Metformin Cerebrolysin Vitamin D
Regenerative treatments	Neurotrophic factors Suppression of RhoA GTPase DNA vaccine Protein S100B Overcoming the glial scar Stem cell therapy Nanoparticles
Treatment based on physical principles	Hyperbaric oxygen therapy Non-invasive brain stimulation
Complementary therapy	Phytotherapy Acupuncture
Mild TBI management	

## Data Availability

No new data were created or analyzed in this study. Data sharing does not apply to this article.
